# INTRODUCING A SERVICE DELIVERY MODEL TO OPTIMIZE HOME MODIFICATION OUTCOMES

**DOI:** 10.1093/geroni/igae098.0010

**Published:** 2024-12-31

**Authors:** Emily Nabors, Kathleen Cameron, Mengzhao Yan, Jon Pynoos, Scott Trudeau

**Affiliations:** National Council on Aging, Arlington, Virginia, United States; National Council on Aging, Arlington, Virginia, United States; University of Southern California, Los Angeles, California, United States; University of Southern California, Los Angeles, California, United States; American Occupational Therapy Association, Stow, Massachusetts, United States

## Abstract

Home modification services have become more widely available in response to the aging population’s growing need for accessible home environments. Despite this growth, the home modification service delivery process is not well-defined, leading to services that do not adequately meet the needs of older adults. To address this gap, the Service Delivery Subcommittee of the National Home Safety and Home Modification Work Group, led by the National Council on Aging and funded by the Administration for Community Living, developed a Home Modification Service Delivery Model. Using an iterative, consensus building approach, this group of interdisciplinary practitioners and researchers developed a model to describe the ideal home modification service delivery process based on evidence and expert perspectives. The model outlines the value of working in a transdisciplinary team, describes key providers and professionals to involve on the team, and their respective roles in the home modification service delivery process (i.e., intake, assessment, completion of modifications, and follow-up). An understanding of the complexity of home modification and awareness of the types of providers that can and should be involved across all phases of home modification can increase the likelihood that consumers will receive the right services at the right time to adequately meet their current and future needs, regardless of their background and point of entry to the process. The Home Modification Service Delivery Model sets a critical foundation for future research and implementation efforts in the community. This session will review the model, describe the development process, and highlight next steps.

